# Investigating Fast Mapping Task Components: No Evidence for the Role of Semantic Referent nor Semantic Inference in Healthy Adults

**DOI:** 10.3389/fpsyg.2019.00394

**Published:** 2019-03-19

**Authors:** Elisa Cooper, Andrea Greve, Richard N. Henson

**Affiliations:** Medical Research Council Cognition and Brain Sciences Unit, University of Cambridge, Cambridge, United Kingdom

**Keywords:** fast mapping, episodic encoding, hippocampus, learning, memory

## Abstract

Fast mapping (FM) is an incidental learning process that is hypothesized to allow rapid, cortical-based memory formation, independent of the normal, hippocampally dependent episodic memory system. It is believed to underlie the rapid vocabulary learning in infants that occurs separately from intentional memorisation strategies. Interest in adult FM learning was stimulated by a report in which adults with amnesia following hippocampal damage showed a normal ability to learn new object-name associations after an incidental FM task, despite their impaired memory under a conventional intentional memorization task. This remarkable finding has important implications for memory rehabilitation, and has led to a number of neuropsychological and neuroimaging studies in other patients and controls. Given this growing interest in adult FM, we conducted four behavioural experiments with healthy adults (*N* = 24 young or older adults in Experiments 1–3 using within-participant designs; *N* = 195 young adults in Experiment 4 using a between-participant design) that attempted to dissect which component(s) of the FM task are important for memory. Two key components of the FM task have been claimed to support FM learning: (1) provision of a known semantic referent and (2) requirement that the new association be inferred. Experiment 1 provided no evidence that removing the semantic referent impaired memory performance, while Experiment 2 provided no evidence that removing the semantic inference impaired performance. Experiment 3 was a replication of Experiment 2 with older participants, based on the hypothesis (from studies of amnesic individuals) that FM would be more effective following the hippocampal atrophy typical of increasing age, but again found no evidence that semantic inference is beneficial. Given potential concerns about contamination between tasks when each participant performed multiple variants of the FM task, we ran a final between-participant design in which each participant only ever did one condition. Despite 80% power and despite being able to detect better memory following intentional memorization in the explicit encoding (EE) control condition than in each of the FM conditions, we again found no evidence of differences between any FM conditions. We conclude that there is no evidence that the components hypothesized to be critical for FM are relevant to healthy adults.

## Introduction

A prevailing theory in the cognitive neuroscience of memory is that the hippocampus supports the rapid acquisition of new information that is subsequently consolidated over time into the neocortex for longer term storage (Norman and O’Reilly, 2003; Squire and Bayley, 2007; Mckenzie and Eichenbaum, 2011). A great deal of interest was therefore generated from a study by Sharon et al. (2011), which investigated four individuals with amnesia following hippocampal damage. Though impaired, as expected, in their ability to learn new object names after standard intentional learning (the explicit encoding or EE condition), these individuals could learn as well as controls after a “fast mapping” (FM) learning procedure, on both immediate and delayed tests of explicit, associative memory. This is an amazing finding, not only in suggesting rapid cortical learning bypassing the hippocampus, contrary to standard theory, but also because of the translational possibility of recovering memory in individuals with hippocampal damage.

The FM task developed by Sharon et al. (2011) was inspired by the concept of “fast mapping” in the developmental literature, namely how infants rapidly acquire vocabulary from relatively few, incidental learning exposures (Sharon et al., 2011; for reviews, see Carey, 2010; Swingley, 2010). For example, infants are able to associate a phonological label, as well as partial syntactic and semantic information, with an unknown object (Carey, 1978; Carey and Bartlett, 1978; Heibeck and Markman, 1987; Bloom, 2001; Katz et al., 1974), even when these associations must be inferred from relating continuous adult speech to the infant’s environment (i.e., without explicit instruction to learn the new name). It is believed that infants deduce what item is being referenced (Heibeck and Markman, 1987; Markman, 1990; Bloom and Markson, 1998; Spiegel and Halberda, 2011) by (implicitly) activating semantic information about similar, known objects, and by excluding other objects in the environment that they already know (Carey, 1978; Carey and Bartlett, 1978; Bloom and Markson, 1998). The FM task, which was devised by Sharon et al. (2011), was designed to mimic this infant experience, so that learning is not only incidental but also involves two key components: (1) semantic features that are shared between the new, unknown object and a second object that is already known and (2) a disjunctive semantic inference that allows the referent of the new name to be determined.

**Figure 1:**
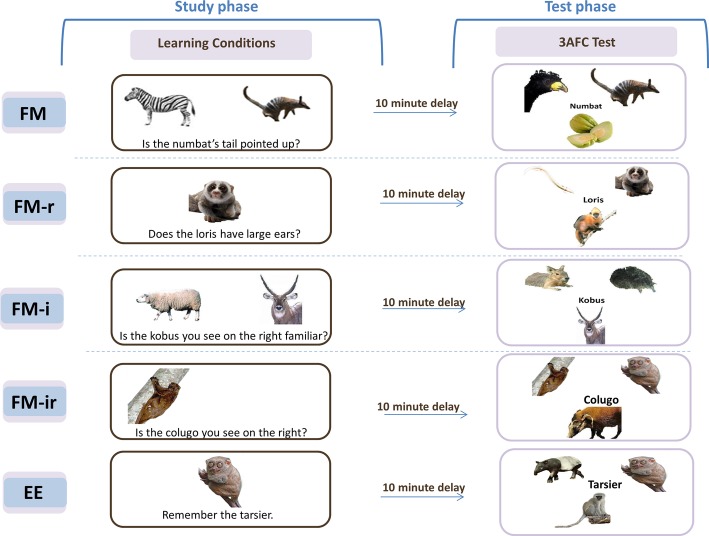
Original fast mapping (FM) procedure, FM variants, and explicit encoding (EE) conditions. Five conditions were implemented across four experiments. Experiments 1–3 tested three of these conditions, in separate study-test blocks, in within-participant designs. Experiment 4 tested all five conditions in a between-participant design. Experiment 1 used conditions FM, FM-r, and FM-ir; Experiment 2 was FM, FM-i, and FM-ir; Experiment 3 replicated the conditions and methods of Experiment 2, but in a healthy older group; Experiment 4 implemented FM, FM-r, FM-i, FM-ir, and EE in between-participants in young groups. For additional information on learning conditions, see main text. In the study phase for FM variants, names were to be incidentally associated with the unknown picture. Key prompts for “yes”/“no” were displayed at the bottom of the screen, on respective sides, but are omitted from this figure for simplicity. In the EE condition (administered in Experiment 4 only), participants were instructed to learn. The test phase, which was methodologically identical in all experiments and conditions, explicitly tested memory in a 3 Alternative Force Choice (3AFC) test. Study and test phases were separated by a 6*–*10 min nonverbal task. In Experiments 1–3, presentation order of learning condition and stimuli set-to-condition assignment was counterbalanced across participants within each experiment.

Figure 1 shows an example trial of Sharon et al.’s FM condition. A picture of an unknown object (a rare animal or fruit) was presented simultaneously with a picture of a known object (semantic referent), and the name of the unknown object was inferred from a question that pertained to the two objects (e.g., “Is the numbat’s tail pointed up?”; the semantic inference). In the more conventional EE control condition, the single unknown object and name were presented simply with instructions to learn them. Memory was later assessed by asking participants to match each name to one of the three objects (a three alternative forced choice test, 3AFC). Individuals with hippocampal damage performed worse than controls in the EE condition, as would be expected, but amazingly their performance in the FM condition did not differ from controls. Indeed, they showed better memory under the FM than EE condition, unlike the controls that showed the opposite pattern. Sharon et al. suggested that the FM learning condition allows rapid, cortical learning that emerges in individuals with hippocampal damage, but is masked by hippocampal-dependent learning in healthy individuals (which is assumed to apply to both conditions). This landmark study triggered a growing interest in FM in adults (see Cooper et al., 2018, for a review). The question of what ingredients of the FM task are critical for learning is theoretically important, in order to understand its potential mechanisms. The literature provides some hints. For example, the role of semantic information, as provided by the referent object, is suggested by Sharon et al. (2011) to play an important role. They report that the FM procedure did not benefit a second group of patients with anterior temporal lobe (ATL) damage, a region that has been associated with semantic processing (Patterson et al., 2007). This is also consistent with the results of Experiment 2 of Coutanche and Thompson-Schill (2014), in which a semantic referent was excluded one of the learning conditions. Eliminating the semantic referent removed evidence of any implicit measure of learning that was present in their full FM condition. Other evidence is less clear however. If semantic information triggered by the known referent is important for FM learning, then one might expect that the more typical the known item, the more its semantic features should overlap with the unknown item, and hence the greater the FM advantage. Yet Coutanche and Koch (2017) reported the opposite, with evidence for implicit memory only when the known referent item was more *atypical*.

In terms of the role of the semantic inference, Warren and colleagues (Warren and Duff, 2014; Warren et al., 2016) did not include a question about a feature of the new item (they only had to click on the unfamiliar item). This lack of semantic inference may explain why they failed to replicate the FM advantage in individuals with memory difficulties reported by Sharon et al. (2011). Yet to our knowledge, no study has directly compared FM conditions with and without the requirement to infer semantic features about the unknown item.

To investigate these key features of FM, we systematically stripped back the components of the FM task through four FM variants across four experiments on young and older healthy volunteers. Experiments 1–3 used within-participant designs; whereas Experiment 4 used a between-participant design to ensure all FM variants remained incidental in nature. We tested the differences in these conditions using an explicit test of memory (using the same 3AFC used in most previous studies), rather than an implicit test like Coutanche and Thompson-Schill (2014), given that an explicit test of memory better captures what would be most relevant to potentially help people with memory problems.

Since a consistent pattern from the literature is that the intentional encoding of the EE condition produces better explicit memory than the incidental encoding of the FM condition in healthy adults (Cooper et al., 2018) and we did not want to encourage intentional encoding strategies in the FM conditions, we did not include an EE condition in the within-participant designs (Experiments 1–3). However, we did add an EE condition in the between-participant design of Experiment 4. This is important to demonstrate that we had sufficient power to detect the advantage of EE over FM conditions in healthy adults that is consistently reported in previous studies. Moreover, this EE advantage in healthy people (contrary to the FM advantage in people with hippocampal amnesia) is important, because it suggests that performance in the FM conditions in healthy people is not completely masked by (hippocampally dependent) explicit encoding mechanisms (otherwise EE and FM performance would be comparable). Therefore, to the extent that any hypothetical fast mapping processes contribute to performance in the FM conditions and these processes depend on a semantic referent and/or semantic inference, then we should still see differences between memory performance in those FM conditions.

In summary, the four variants of FM were (Figure 1): (1) the original FM procedure, which presents pictures of two objects – one known (the semantic referent) and one unknown (whose name is to be learned) – and a question that requires the participant to infer the name of the unknown object (FM), (2) a variant still requiring a semantic inference but without the semantic referent (FM-r), (3) a variant with a semantic referent but no semantic inference (FM-i), and (4) a variant with no referent or inference about the object name (FM-ir). If only the semantic referent is required (e.g., to activate pre-existing knowledge or a schema; Sharon et al., 2011; Coutanche and Thompson-Schill, 2014), then performance on the explicit memory test should be FM = FM-i > FM-r = FM-ir. If only a semantic inference is required (e.g., to engage more elaborative encoding; Craik and Tulving, 1975), then performance should be FM = FM-r > FM-i = FM-ir. Other alternatives are that both make independent contributions (FM > FM-r = FM-i > FM-ir) or that their conjunction is necessary (FM > FM-r = FM-i = FM-ir).

## Experiment 1

In Experiment 1, we investigated whether the semantic referent improved subsequent memory when learning a new object name, i.e., tested three of the four conditions in Figure 1: the original FM procedure (FM), a condition that removes the known referent item (FM-r) and a condition that removes the known referent and the semantic inference question (FM-ir). As suggested by Sharon et al. (2011), and supported (for implicit memory) by Coutanche and Thompson-Schill (2014), a semantic referent might be important to link the newly acquired object name to existing knowledge about related objects (e.g., to compare and contrast a new animal called a “numbat” to a similar known animal, the zebra). We tested all three conditions in young participants in a within-participant design.

### Materials and Methods

#### Participants

Twenty-four young (aged 18–40, 18 females) volunteers were recruited from the MRC Cognition and Brain Sciences Unit’s Volunteer Panel, provided written informed consent prior to taking part and were compensated financially for their time. They reported being native British English speakers, having normal or corrected-to-normal vision, and no difficulties with their hearing. Their inclusion was approved by the Cambridge Psychological Research Ethics Committee (reference 2005.08) and procedures accorded with the Declaration of Helsinki.

#### Stimuli

Stimuli were 144 color photographs and corresponding names of real animals, plants, flowers, fruits, and vegetables, of which 72 were unknown items, and 72 were known items from the same categories as the unknown stimuli. The 72 unknown, but real items were an intermixed set of Greve et al. (2014) 48 culturally normed, unknown items, and an additional 24 culturally normed unknown items. These were divided into 3 sets of 24 picture-with-name items and were paired with 24 known items from the same category. Each participant received all three sets, with each set appearing in one of the three study conditions: FM, no known referent (FM-r), and no known referent and no feature inference (FM-ir). Set-to-condition assignment was counterbalanced across participants. Known item pictures appeared in FM and their names were never used. Only the studied, unknown items from the corresponding study section were used in the 3 Alternative Force Choice (3AFC) test.

Note that there were insufficient stimuli to run all 4 FM variant conditions within the same experiment, while maintaining control of the stimulus properties (plus there were time constraints on the within-participant design). Later, we compare all four conditions, in between-participant comparions, by pooling first blocks across Experiments 1–3, and in a fully between-participant design in Experiment 4.

#### Procedure

The paradigm is shown in Figure 1. E-prime 2 (Psychological Software Tools Inc., 2012) was used to display stimuli and collect button press responses. Participants completed three study-test phases, one per condition. Order of conditions within the session was counterbalanced across participant. Presentation order was crossed with set (Set 1, Set 2, and Set 3) and variant learning condition (FM, FM-r, or FM-ir), resulting in a counterbalance of six that was repeated four times. Due to a procedural error, one counter balance was run twice, leading to a slightly uneven number of participants per counterbalance, N = 7, 8, and 9.

The variant learning conditions, whether FM, FM-r, or FM-ir, occurred in the study phase during which participants incidentally learned the real names of unknown items. There was a 10-min delay filled with a nonverbal task (see below) between study and test phases. Test phase procedures were identical regardless of the study phase. In the test phase, explicit memory for the items’ names was tested in a 3AFC recognition memory test. Participants sat 66 cm in front of a LCD 48.26 cm (19 inch) computer monitor. All items were displayed on a screen with a white background, and text and fixation crosses were black 24 point bold Arial font, except for button-response prompts which were in grey.

##### Study Phase

There were three learning conditions. FM, FM-r, or FM-ir, each made up one study phase of the three study-test blocks. In all conditions, learning was designed to be incidental, and participants were informed that this was a visual object perception experiment; they should answer the yes/no question about the picture(s) using keys on the left and right of the keyboard. They were not informed of later memory tests (and only study trials were presented in the practice), though they may have guessed this in later blocks through prior experience; an issue that we address in Experiment 4.

In the FM condition, the original fast mapping learning procedure was used, and it contained both a picture of semantic referent and a question requiring disjunctive inference about a feature. Two pictures appeared on the screen, one known and the other unknown but from the same category, with a yes/no question about a feature (e.g., “Is the numbat’s tail pointed upward?”), with a different orienting question for each of the two appearances of the unknown item (see below). The other variant learning conditions systematically removed task components. The FM-r condition contained a question about a feature, but the known referent was removed (only the unknown picture appeared). In the FM-ir condition, the known referent was not presented, and the question required little visual inspection and no feature inference (e.g., “Is the numbat you see on the right?” when there was only one picture on the screen). Note that the FM-ir condition (and FM-i condition in Experiments 2–3) does require an inference, but not one that related to the semantic features of the object.

Each study phase started with a white screen for 500 ms. This was followed by the stimuli, which were displayed for a fixed 6.5 s. First, the question was presented visually on the screen and aurally over headphones; the stimuli picture(s) were added to the visual presentation, and participants answered the question by pressing a response key. This cycle continued until all 24 unknown stimuli had been viewed twice. The list of 48 items was presented in random order. In FM, a known (e.g., zebra) and unknown picture (e.g., numbat) were displayed to the left and right of the centre of the screen. For the two presentations, unknown items were paired with a different known item. In the FM-r and FM-ir conditions, only the unknown picture was displayed. In all conditions, a yes/no question appeared below the pictures. For FM, FM-i (used in Experiments 2 and 3) and FM-ir, unknown items appeared once on each side (central-left or central-right) and once with each response type (“yes” or “no”). For FM-r, the picture appeared in the centre of the screen. Prompts for key assignments appeared in the lower-left and lower-right corners of the screen for the respective response and an on-screen prompt appeared if no response was given within 6 s. In all variant learning conditions, the question included the unknown item’s real name (e.g., “numbat”). Prior to each study phase, participants completed a separate run of seven practice study trials (relevant to the impending learning condition) with feedback and unique stimuli. There was no practice of the test phase(s).

##### Test Phase

The study phase and test phase were separated by a 10-min delay during which participants performed a nonverbal task to prevent rehearsal, which was a test of general intelligence “g” as measured by the Cattell Culture Fair Scale 2 intelligence test (Cattell and Cattell, 1960). In each test phase, memory for the unknown items’ names from the preceding study phase was measured with a 3AFC recognition memory test, as expanded below.

In the 3AFC test, each of the 24 studied names was presented centrally between three unknown pictures from the study phase, one at the top-left, one at the top-right, and one at the central-bottom of the screen. Participants indicated which picture matched the name by using one of three keys, with each of the three locations corresponding to the correct response approximately equally often. Each unknown item picture was displayed three times, once in each location, once as the correct answer and twice as a foil. Trials were displayed in a random order, remained on the screen until a response was given, and were separated by a 500-ms white screen.

After the test phase, participants completed a familiarity test, in which they were shown each unknown picture once more and reported whether or not they were familiar with it prior to the experiment. The plan was to exclude these “already familiar” items from the analysis, though due to experimenter error, these data were lost for Experiment 1 (though see Experiments 2 and 3).

#### Analyses

Data from all experiments were analysed using R. Experiments 1–3 were analysed using version 3.2.5, and Experiment 4 was analysed using 3.5.0 (R Core Team, 2015, 2018, respectively). The measure of learning was the proportion correct (accuracy) in the 3AFC memory test, with chance being 0.33. In Experiments 1–3, accuracy was analysed in a repeated measures ANOVA with a within-participant factor of variant learning conditions (FM, FM-r, or FM-ir), using a Greenhouse-Geisser correction and post-hoc paired *t*-tests. ANOVAs were performed using the “ez” package in R (Lawrence, 2016). *p*’s are two tailed, unless otherwise reported, with alpha set at 0.05. Bayes factors were calculated using the “BayesFactor” package in R (Morey et al., 2018). The R script and data are available on https://osf.io/3mpnw/.

### Results

**Figure 2:**
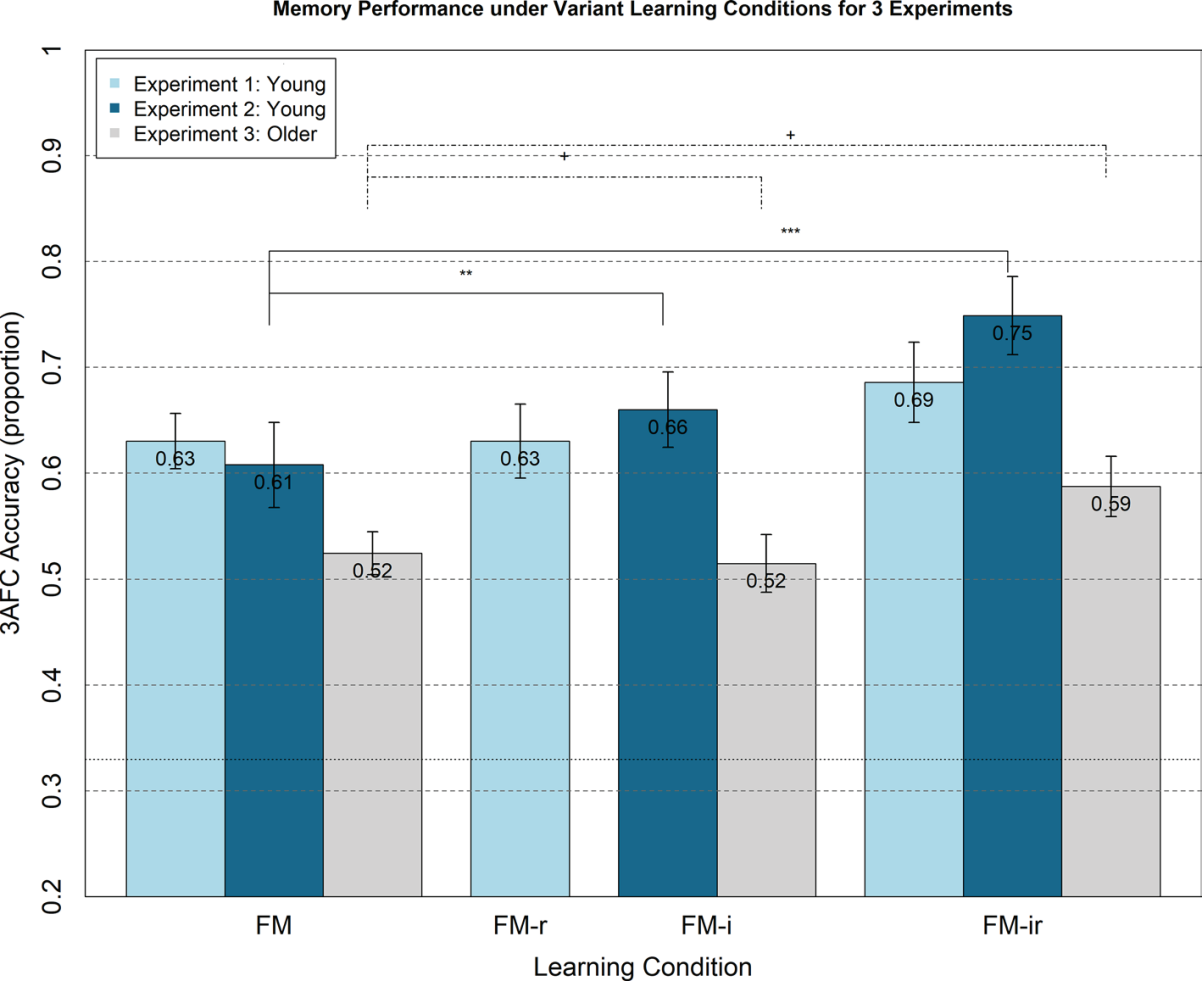
Memory performance in Experiments 1–3 from 3AFC at the test phase (chance = 0.33) for FM variants administered within-participant. Experiment 1 (light blue bars) shows memory performance for healthy young adults under FM, FM-r, and FM-ir; there were no significant differences between conditions. Experiment 2 (dark blue bars) shows memory performance for a different group of healthy young adults under FM, FM-i, and FM-ir. Significant differences are marked ** = *p* < 0.01, *** = *p* < 0.001. Experiment 3 (grey bars) shows performance of healthy older adults under same conditions as Experiment 2. Trending differences are marked + = p < 0.064. Mean performance is reported at the top of each bar. Error bars are standard error of the mean.

The ANOVA on mean 3AFC accuracy performance showed no significant main effect of condition, *F*(1.91, 43.92) = 1.39, *p* = 0.26; see light blue bars in Figure 2. Post-hoc, paired *t*-tests for FM versus FM-r, FM-r versus FM-ir, and FM versus FM-ir revealed the same nonsignificant pattern, all *t*(23)’s < 1.52, all *p*’s > 0.143. In other words, there was no evidence that memory performance differed between conditions.

Given that the absence of evidence is not evidence of absence, three additional analyses were performed to provide a Bayes factor for each paired comparison. A one-sided, paired Bayesian *t*-test with a Cauchy prior scaled at sqrt(2)/2 (medium scaling) compared the null hypothesis versus the predicted hypothesis that memory performance under each pair of FM variants differed (Rouder et al., 2012). For the FM versus FM-r comparison, the null hypothesis was supported with a Bayes factor of 4.66, while for the FM versus FM-ir comparison, the null hypothesis was supported with a Bayes factor of 9.76. Only for the FM-r versus FM-ir comparison was there insufficient evidence for either hypothesis, with a Bayes factor for the null hypothesis of 0.93. Thus, there was moderate to strong evidence against the predicted hypothesis that removing the semantic referent impairs memory.

**Figure 3:**
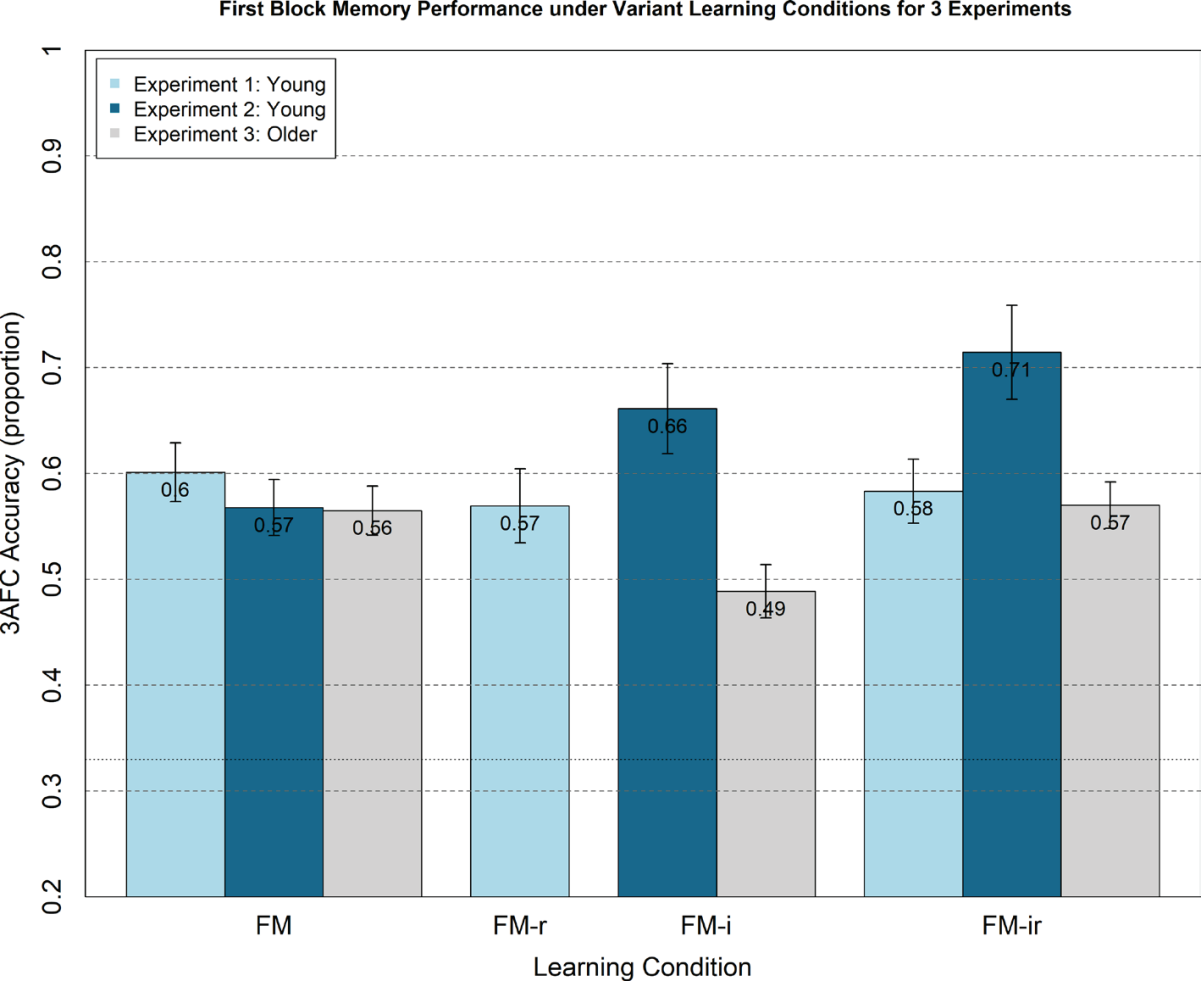
Analogous plot to Figure 2, except 3AFC memory test performance (chance = 0.33) is from the first study-test block only, for which participants were not aware of test phase.

Though not informed of the later memory tests, participants would become aware of the memory component after the first block. This may have caused them to adopt an intentional encoding strategy in subsequent blocks, which could reduce differences between the three conditions. Furthermore, there may be carry-over effects from attempting one version of the FM task on performance of subsequent versions. We therefore examined performance on just the first study-test block, during the study of which participants were not aware their memory would be tested, nor of other variants of the FM task. A one-way, between-participant ANOVA still showed no significant main effect of FM variant, *F*(2, 21) = 0.08, *p* = 0.92, nor were any *t*-test differences significant, *t*(14) < 0.40, *p* > 0.70 (Figure 3).

### Discussion

Experiment 1 found no evidence of any difference in explicit memory performance across three variants of the FM procedure: FM, FM-r, and FM-ir. This suggests that a semantic referent is not important to link the newly acquired object name to existing knowledge about related objects (cf., Sharon et al., 2011; Gilboa and Marlatte, 2017). Though we did not even find evidence for a significant difference between the two extreme cases (FM and FM-ir), it is still possible that the requirement to infer the name of the unfamiliar object is important for deeper encoding (Craik and Tulving, 1975) and we simply failed to detect a difference between FM and FM-ir conditions (despite the numerical trend for FM-ir > FM). Experiment 2 therefore replaced the FM-r condition of Experiment 1, which removed the known referent, with the FM-i condition (Figure 1), which removed the semantic inference question, and repeated the FM and FM-ir conditions in a new group of young participants.

## Experiment 2

Experiment 2 was identical to Experiment 1 except the FM-r condition was replaced with the FM-i condition, in which a semantic referent was present, but there was only a simple question that did not require processing any semantic features of the unknown object (Figure 1). If such “semantic elaboration” is important for learning, then one would expect memory performance to be best under FM, resulting in FM > FM-i = FM-ir.

### Materials and Methods

#### Participants

Twenty-four young (aged 19–40, 13 females) volunteers, different from those in Experiment 1, were recruited using the same procedure as Experiment 1.

#### Stimuli and Procedure

Stimuli and procedure were identical to Experiment 1 with the exception that the FM-i condition replaced the FM-r condition (Figure 1), i.e., the three different study phases were: FM, FM-i, and FM-ir with FM and FM-ir being identical to Experiment 1. In FM-i and FM-ir conditions, the question could be answered without processing semantic features of the unknown object.

#### Analyses

One difference from Experiment 1 is that we were able to measure how many of the critical objects that were supposed to be unknown were in fact familiar to participants before starting the experiment. The average number of such items was 1.21 (out of 24 per condition). Trials with these pre-experimentally familiar items were removed from the analyses below, but their low number (consistent with our prior work with these stimuli; Greve et al., 2014) suggests that their inclusion in Experiment 1 is extremely unlikely to affect its results.

### Results

Means and standard errors per condition are shown in Figure 2. Unlike Experiment 1, there was a significant main effect of condition, *F*(1.74, 40.08) = 10.62, *p* < 0.001. However, the means showed the opposite pattern to the prediction, namely worse memory as components of the FM procedure were removed. Paired *t*-tests showed that performance was significantly *better* in the FM-ir than FM-i condition and in the FM-ir condition than FM condition, *t*(23) = 3.46, *p* < 0.01 and *t*(23) = 4.66, *p* < 0.001, respectively. Performance in the FM and FM-i conditions did not differ significantly, *t*(23) = 1.45, *p* = 0.16. In other words, the pattern of significant differences was FM = FM-i < FM-ir.

For completeness, to match Experiment 1, we calculated Bayes factors for each pairwise comparison. For the FM versus FM-i comparison, the Bayes factor was 10.30 in favor of the null; for the FM-i versus FM-ir comparison, it was 17.03 in favor of null, and for the FM versus FM-ir comparison, it was 19.70. Thus, there is strong evidence that the null hypothesis is more likely than the predicted hypothesis that removing components of FM impairs performance.

As with Experiment 1, we analysed first study blocks only to rule out any order effects, e.g., participants adopting intentional learning strategies or experiencing carry-over effects from doing one FM condition on another. A between-participant ANOVA showed no significant main effect of condition, *F*(2, 21) = 1.23, *p* = 0.31, and none of the *t*-tests reached significance, *t*(14) < 1.64, *p* > 0.12, though the numerical pattern showed the same increase, rather than decrease, from FM to FM-ir (Figure 3).

### Discussion

Experiment 2 found no evidence that removing the requirement for a semantic inference on the unknown object impaired memory performance. In fact, when this inference was removed, along with the semantic referent (FM-ir condition), memory was actually better rather than worse. This could reflect the fact that the lack of semantic referent and inference meant that the FM-ir condition had least “cognitive load,” given that increased load at encoding tends to impair memory (e.g., Craik et al., 1996). However, this interpretation should be treated with caution, because the same comparison (FM-ir versus FM) was not significant in Experiment 1, nor did it remain significant in Experiment 2 when only the first block was analysed to control for order effects (though the numerical pattern remained). Even so, Experiment 2 is consistent with Experiment 1 in providing no support that two components that have been hypothesized as essential for fast mapping are, in fact, critical.

To bolster our results, we repeated Experiment 2 with an older group of healthy adults. It is possible that, in the presence of an intact episodic memory system (supported by hippocampus), any independent fast mapping processes (acting directly in neocortex) are masked – i.e., performance across our FM variants is equivalent because it is dominated by episodic memory. Given evidence that episodic memory and hippocampal volume, both decline with healthy aging (e.g., Greve et al., 2014), one might expect differences between FM variants to emerge in an healthy older group.

## Experiment 3

Experiment 3 was a replication of Experiment 2, except that the volunteers were older (59–76 years) than the young group. If age reduces the influence of episodic memory, due to healthy reduction in hippocampal volumes, then the important components of FM should become more apparent, i.e., FM > FM-ir (and possibly FM > FM-i).

### Materials and Methods

#### Participants

Twenty-four volunteers (aged 59–76, 11 females), who were older than the “young groups,” in Experiments 1–2 were recruited using the same procedure as Experiment 1.

#### Stimuli, Procedure, and Analysis

All stimuli, design, procedural, and analysis details were identical to Experiment 2. Only items that were not pre-experimentally familiar were included in the analyses. The average number of pre-experimentally familiar items was 1.35.

### Results

#### Experiment 3

Any main effect of FM or FM variant learning condition did not reach significance, *F*(1.91, 43.9) = 2.737, *p* = 0.078, but showed a similar numerical pattern to Experiment 2 (see Figure 2), with best memory performance in the FM-ir condition. Indeed, post-hoc paired *t*-tests showed a trend for better performance in the FM-ir condition than FM or FM-i conditions, *t*(23) = 2.01, *p* < 0.056 and *t*(23) = 1.95, *p* < 0.063, respectively. Performance in the FM and FM-i conditions did not differ significantly, *t*(23) = 0.30, *p* = 0.77.

For the FM versus FM-i comparison, the Bayes factor was 3.67 in favor of the null; for the FM-i versus FM-ir comparison, it was 12.24 in favor of null, and for the FM versus FM-ir comparison, it was 12.45. Thus again, there was strong evidence that the null hypothesis is more likely than the predicted hypothesis that removing components of FM impairs performance.

We again examined possible effects of presentation order (which was counterbalanced across participants), by analyzing memory performance in the first block only. The ANOVA showed no main effect of condition for the first study-block only, *F*(2, 21) = 1.26, *p* = 0.31, and *t*-tests showed no difference between conditions, *t*(14) < 1.41, *p* > 0.18.

#### Combined Analysis With Experiment 2

Given the identical designs, we entered the data from both Experiment 2 and Experiment 3 into a mixed ANOVA with an additional between-participant factor of age group (young and older), in order to confirm effects of age and test for any interactions between age group and FM variant condition. The main effect of age was significant with the expected worse performance for the older group, F(1, 46) = 12.33, *p* = 0.001 (M = 0.67 vs. 0.54, young versus older, respectively), but there was no significant interaction between group and FM condition, *F*(2, 92) = 1.62, *p* = 0.20. The effect of condition was now significant, F (2, 92) = 11.07, *p* < 0.001, with best memory performance under FM-ir, then FM-r, and worst under FM, again opposite to the predictions of the fast mapping account, but potentially compatible with an alternative hypothesis of increased cognitive load from FM to FM-ir.

### Discussion

The pattern of means for the older group tested in Experiment 3 was very similar to that for the young group tested in Experiment 2, with no evidence that removing components of the FM procedure impaired performance. In fact, when combining the two experiments, the main effect of learning condition became significant, but memory was best when both components were absent. Indeed, there was no evidence that the pattern differed with age (the interaction in the combined analysis across both experiments was not significant), despite the older group doing worse overall, as expected with the frequently found impairments of explicit memory with healthy aging.

## Combined Analysis Across Experiments 1–3

In order to maximize power, we re-parametrized the design of Experiments 1–3 according to the number of components removed – either 0 (FM-0, i.e., FM), 1 (FM-1, i.e., FM-r or FM-i), or 2 (FM-2, i.e., FM-ir) – and entered all 72 participants (regardless of age). There was a significant main effect, *F*(1.99, 141.2) = 10.85, *p* < 0.001, with the pattern of means increasing as components were removed (M = 0.59, 0.60, and 0.67, respectively), though paired *t*-tests showed than only the FM-2 condition was significantly better than the other two conditions, *t*(71) > 3.44, *p* < 0.005. In other words, the pattern of means was FM-0 < FM-1 < FM-2, consistent with decreasing cognitive load improving memory, and opposite to the pattern predicted from the fasting mapping hypothesis of fewer FM components impairing memory.

When analyzing first study-test blocks only (using a between-participant ANOVA), the means showed a similar numerical increase as more components were removed (M = 0.57, 0.59, and 0.61, respectively). However, the main effect of FM condition was no longer significant, *F*(1, 67) = 0.99, *p* = 0.32, nor were any of the *t*-test comparisons, *t*(45) < 1.05, *p* > 0.298. This could reflect the reduced statistical power of this between-participant design, or could reflect real effects of block order, such that the first block was more incidental in nature than later blocks. To tease apart these two possibilities, we conducted a final experiment in young adults with a fully between-participant design, in which each participant only completed one task block (so there could be no carry-over effect across blocks). Importantly, we powered this final experiment to have an 80% probability of detecting a difference between the two extreme FM variants (FM vs. FM-ir) at least as big as that found when combining the young participants in Experiments 1 and 2 (even though that difference was in the opposite direction to that predicted by the fast mapping hypothesis and in line with the alternative prediction of a cognitive load hypothesis).

## Experiment 4

Experiment 4 included all five conditions in Figure 1, i.e., the 4 FM variants (FM, FM-i, FM-r, and FM-ir) plus the original explicit encoding (EE) condition of Sharon et al. (2011). Each condition was administered to a separate group of 39 participants in order to minimize the likelihood of intentional memorization strategies in the FM conditions. The addition of the EE group also served to ensure we had sufficient sensitivity to replicate the well-established finding of superior memory in the EE condition than FM conditions (Cooper et al., 2018).

### Materials and Methods

#### Participants

One-hundred and ninety-seven young (aged 18–40) participants were recruited through Prolific,^1^ which is a web-based crowd-sourcing platform that can be integrated with online experiments. The final data set was comprised of 195 participants^2^ from a potential pool of 3,789 that met the recruitment criteria below. Participants were randomly allocated to one of five conditions (FM, FM-i, FM-r, FM-ir, or EE) (N = 39 in each group, FM mean age 28.2 years, 22 females; FM-i mean age 30.4 years, 30 females; FM-r mean age 27.9 years, 22 females; FM-ir mean age 29.9 years, 28 females; EE mean age 27.4 years, 27 females). The figure of 39 participants per group was chosen to provide an *a priori* power of over 80% (actual power was 80.23%), given an effect size of 0.57 for a one-sided, between-participant *t*-test based on a comparison of 3AFC performance between the two extreme FM variant conditions (FM versus FM-ir) using data combined across Experiments 1 and 2 (since both used young participants).

All participants provided informed consent electronically, online prior to taking part and were compensated financially for their time. They reported being monolingual English speakers, who were UK citizens currently residing in the UK, since the stimuli were normed for a UK population. Participants also reported having normal or corrected-to-normal vision, no language difficulties, and having never received a diagnosis of autism. They also previously participated in a minimum of two online studies on the Prolific platform with an approval rating at least 90%. Their inclusion was approved by the Cambridge Psychological Research Ethics Committee (reference PRE2016.055) and procedures accorded with the Declaration of Helsinki.

#### Stimuli

All stimuli were identical to previous experiments. As in Experiment 1, there were three stimulus sets (Set 1, 2, and 3). Each set was administered equally under each of the five conditions, such that there were N = 13 participants per set per condition.

#### Procedure

Data collection occurred online on the participants’ computer at a location of the participants’ choice. The experiment (with five conditions in total) was programmed using a free, open-source tool, JsPsych, based in JavaScript^3^. It was hosted on the MRC-CBU servers using free, open-source JATOS^4^. The servers are based on the EU and compliant with data protection and security policies.

##### Study Phase

Study phases for the FM, FM-i, FM-r, and FM-ir study-test blocks are identical to those detailed and used in Experiment 1–3 except that there was no longer an aural presentation of the question. FM variant study phases were intended to be incidental learning conditions. Participants received identical “ruse” information to that used in Experiments 1–3, with on-screen information detailing that they were taking part in a “picture study” that investigated how pictures are perceived and processed, and how the questions on the screen are answered based on this information. They were not informed of the later memory task.

In the study phase of the additional condition, EE, display details were identical to the other learning conditions, with following exceptions. An unknown item was displayed centrally, with its name and instructions to remember it below (“Remember the tarsier.”) for both study phase presentations. Participants in this condition were informed that it was a memory experiment. They were instructed to study and learn the items and names and informed of a later memory test. No overt response was given during the study phase.

Prior to all study phases, participants completed a separate run of 10 practice study trials with unique stimuli. No test phase was included in the practice.

##### Test Phase

The study phase and test phase for each condition was separated by a minimum delay of 6 min, when participants completed three runs of a nonverbal letter-digit substitution task (an online adaptation of this task: https://healthabc.nia.nih.gov/sites/default/files/dsst_0.pdf). The first run lasted 1 min and the two following runs lasted 2.5 min each. Each run was preceded by instructions and six practice trials. This task was designed to be comparable to the Cattell distractor task performed between study and test in Experiments 1–3.

The test phase for all five conditions was identical to the previous experiments, except for a recall memory test prior to the 3AFC memory test. In the recall phase, participants reported recollected names from the initial study phase, *via* typing in a response box, which was presented until participants submitted their responses. Following the test phase, there was a pre-experimental familiarity test identical to Experiments 2 and 3.

#### Analyses

Memory measures were analysed using a one-way, between-participant ANOVA with five conditions (FM, FM-i, FM-r, FM-ir, and EE), followed-up with pairwise, independent sample *t*-tests (FM versus FM-r, FM-i, FM-ir, and EE; FM-r versus FM-i, FM-ir, and EE; FM-i versus FM-ir and EE; FM-ir versus EE). As in Experiments 2 and 3, we measured how many critical objects that were supposed to be unknown were in fact familiar to participants before starting the experiment. There average number of pre-experimentally familiar items was 2.1 (out of a maximum of 24), and these were not included in the analyses^5^. Other details are identical to those detailed in Experiment 1.

### Results

The median number of items recalled was 0 in all FM conditions and 4 in the EE condition (out of a maximum of 24). Since these data were near floor, they were analysed using a nonparametric Kruskal-Wallis ANOVA. There was a significant main effect of condition (*X* ^2^ = 69.50, *df* = 4, and *p* < 0.001), but between-participant pairwise Mann-Whitney U-tests showed that this was driven by the EE condition, since each FM variant produced worse recall than the EE condition (all *p*’s < 0.001). The only significant difference between FM variants was higher median recall for FM versus FM-r (*p* = 0.047), but the mean ranks for FM, FM-i, FM-r, and FM-ir were 1.27, 0.83, 0.86, and 1.22, respectively, which is not the monontically decreasing pattern predicted by the fast mapping hypothesis, and the significance of this FM versus FM-r difference could be a type I error given the 10 pairwise tests performed.

**Figure 4:**
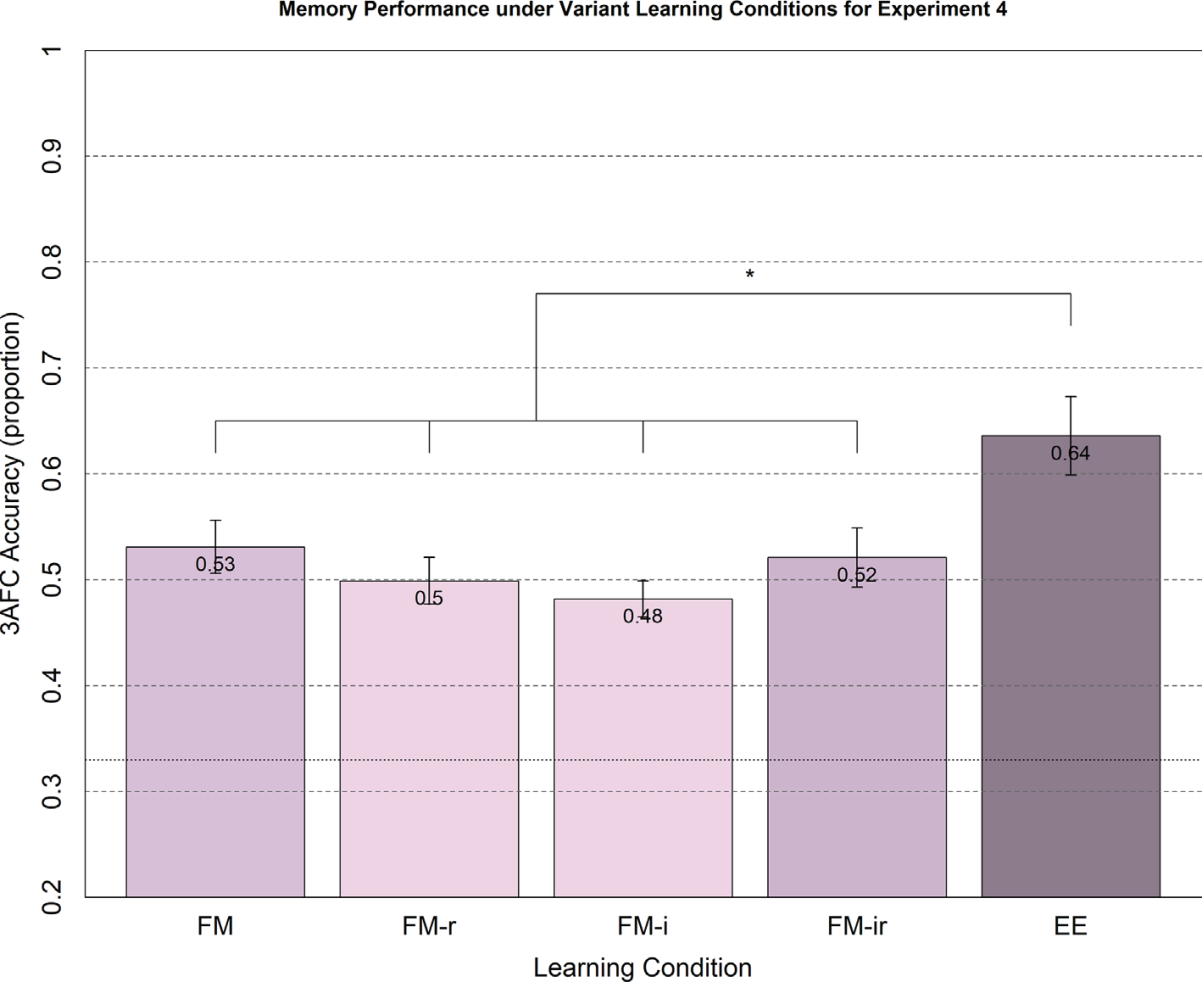
Memory performance in Experiment 4 from 3AFC at the test phase (chance = 0.33) for FM variants and EE. Conditions were administered between-participant. Memory performance for healthy young adults under FM, FM-r, FM-i, and FM-ir did not significantly differ between conditions. All FM conditions significantly differed from the explicit learning condition, EE. Significant differences are marked * = p < 0.05, two tailed. Mean performance is reported at the top of each bar. Error bars are standard error of the mean.

The means and standard errors for the main test of 3AFC performance are shown in Figure 4. Memory performance on average was lower than in Experiments 1–3, which may reflect the absence of concurrent auditory input at study, a different population recruited, reduced motivation when participating online in the absence of an experimenter and/or the benefit of intentional encoding strategies triggered by the within-participant designs of Experiments 1–3. The parametric ANOVA showed a significant main effect of condition, *F*(4, 190) = 5.05, *p* < 0.01, but this was again driven by the EE condition, since none of the *t*-tests across FM conditions reached significance, *t*(76)’s < 1.62, all *p*’s > 0.11, yet all FM conditions produced significantly worse memory performance than the condition EE, all *t*(76)’s > 2.33, all *p*’s < 0.023.

For consistency with previous experiments, we also calculated Bayes factors. For the two extreme variant conditions, FM versus FM-ir, the null hypothesis was supported with a Bayes factor of 3.46. For the FM-r versus FM-i comparison, the null hypothesis was supported with a Bayes factor of 2.53. For the FM-r versus FM-ir comparison, the null hypothesis was supported with a Bayes factor of 6.34. For the FM-i versus FM-ir comparison, the null hypothesis was supported with a Bayes factor of 8.53. For the FM versus FM-r comparison and FM versus FM-i comparison, there was insufficient evidence for either hypothesis, with a Bayes factor for null of 1.78 and 0.74, respectively. Thus, there was moderate evidence against the predicted hypotheses, including the hypothesis that the removal of the semantic referent and the semantic inference question (FM-ir) impairs memory compared to the full FM task.

### Discussion

Using a between-participant design, Experiment 4 found no evidence that removing components of the FM task impaired 3AFC explict memory performance, despite *a priori* power of over 80% and despite the ability of the experiment to replicate the significant advantage of the EE condition over the FM conditions. Like in Experiments 1–3 and generally confirmed by Bayes Factors, there was no evidence for memory performance decreasing as FM task components are removed, as predicted by the fast mapping hypothesis. There was also no support for the alternative hypothesis developed in Experiments 2–3, that decreasing cognitive load actually improves memory as FM task components are removed. The latter is consistent with the first-block-only results from Experiments 1–3 and suggests that significantly increased memory for the FM-ir condition than FM condition that was found in Experiments 2–3 might only arise when participants perform multiple study-test blocks and therefore adopt intentional memorisation strategies; a point we return to in the General Discussion.

## General Discussion

There is growing interest in the possibility of fast mapping in adults, as a form of incidental learning of novel associations, but to our knowledge, no study has directly tested the psychological components believed to support it, such as the presence of a semantic referent and/or the requirement to infer new semantic information. We took the FM paradigm developed by Sharon et al. (2011), in which the task is to incidentally learn the name of an unfamiliar object (e.g., rare animal or fruit) and successively stripped away (1) the presence of a semantic referent (concurrent picture of a known object) and (2) the requirement for inference (about a semantic feature of the unknown object). In none of our four experiments did we find evidence that memory in healthy adults was impaired when these components were removed from the FM task. Indeed, Bayes factors preferred the null hypothesis of no difference in each experiment. Moreover, in Experiments 2–3, the pattern of means was the opposite to that predicted by the fast mapping hypothesis – i.e., memory was significantly better rather than worse when both FM components were removed – though this opposite pattern did not remain significant when analyzing only the first block and was not significant in Experiment 4, where all conditions were administered between-participant.

What is the reason that removing both FM task components sometimes improved rather than impaired memory in Experiments 2–3, at least when analyzing all blocks? We suggest that the higher cognitive load entailed in the standard FM condition compared to the FM-ir condition (which had neither a semantic referent nor semantic inference) can impair memory encoding (Craik et al., 1996). This reduced cognitive load might also explain why EE performance (with only a single object and name) is always better than FM performance. Moreover, if this load only exerts a detrimental effect when people adopt intentional encoding strategies and such strategies are only adopted after participants realize their memory will be tested following the first study-test block of FM conditions, then this can also explain why the advantage of the FM-ir condition relative to FM condition was not seen when analyzing only the first block in Experiments 1–3, nor in the single study-test block design of Experiment 4.

Regardless of whether learning is incidental or intentional, it is possible that healthy adults benefit from a hippocampally-based explicit memory system, which operates during both EE and FM conditions (Sharon et al., 2011) and which masks differences between the FM variants tested here. The data from Experiment 4, and many prior studies (Cooper et al., 2018), suggest that this hippocampal system does not operate as completely effectively in the FM condition as in the EE condition, since memory in the FM condition is never as good as in the EE condition. Furthermore, based on the prior evidence that the hippocampus shrinks with age, we chose the participants in Experiment 3 to be older, drawn from the same population that we have previously shown to have reduced hippocampal volume (Greve et al., 2014). Yet we still found no evidence of the predicted impairment as components of FM were removed in this older group, and there was no interaction between age and the FM conditions when analyzing across Experiments 2–3. Nonetheless, it remains theoretically possible that differences between FM variants are extremely difficult to detect in either young or older health adults, because memory performance is largely masked by a hippocampal system that operates equivalently in all cases, and (1) the hippocampus has not deteriorated sufficiently with healthy ageing (in contrast to amnesic patients) and (2) any effect of cognitive load on the hippocampal system (as hypothesized above) only arises when encoding is intentional. In this case, the components supporting fast mapping can only effectively be dissected when the hippocampal system is significantly impaired, such as in the patients tested by (Sharon et al., 2011; though see Cooper et al., 2018, for review of other patient studies that find no FM advantage). Repeating the present FM variants in such patients would therefore be an interesting test of this hypothesis.

Finally, it is possible that differences between our FM conditions would be observed by an implicit rather than explicit test of memory, like the reaction times for semantic decisions used by Coutanche and Thompson-Schill (2014) and Coutanche and Koch (2017). However, this requires first replicating these authors’ basic finding of reliable implicit memory after FM but not EE encoding, which we have found difficult to date (Cooper et al., 2018). Nonetheless, on the basis of the present data and that reviewed in the Introduction, we conclude that there is currently no evidence for the critical components hypothesized to underlie fast mapping in adults, at least when those adults are healthy, and memory is tested explicitly.

## Data Availability

The datasets and analysis scripts for this study can be found on the Open Science Framework (OSF), DOI: 10.17605/OSF.IO/3MPNW.

## Author Contributions

EC and AG performed and managed research and data collection for Experiments 1–3. EC managed, programmed, and collected data for Experiment 4, performed analyses for Experiments 1–4 and wrote the initial draft of the manuscript. All authors contributed to experiment design, result interpretation and manuscript revision, and read and approved the submitted version.

## Footnotes

^1^https://prolific.ac/

^2^One was replaced in the FM-i condition due to self-reported dyslexia post-participation. Another was replaced in the EE condition during early data collection.

^3^http://www.jspsych.org/

^4^https://www.jatos.org/

^5^3AFC performance was not corrected for pre-experimentally familiar items for two participants in the FM-r condition. These participants reported being familiar with all stimuli prior to the experiment. Since study and test performance were not at ceiling, this self-report of being able to correctly name all items pre-experimentally was attributed to button mapping error or misinterpreting instructions.
